# Predictability and Resetting in a Case of Convulsive Status Epilepticus

**DOI:** 10.3389/fneur.2018.00172

**Published:** 2018-03-22

**Authors:** Timothy Hutson, Diana Pizarro, Sandipan Pati, Leon D. Iasemidis

**Affiliations:** ^1^Department of Biomedical Engineering, Louisiana Tech University, Ruston, LA, United States; ^2^Department of Neurology, University of Alabama at Birmingham, Birmingham, AL, United States

**Keywords:** status epilepticus, pathological dynamics, predictability, resetting, network connectivity

## Abstract

In this case study, we present evidence of resetting of brain dynamics following convulsive status epilepticus (SE) that was treated successfully with antiepileptic medications (AEDs). The measure of effective inflow (EI), a novel measure of network connectivity, was applied to the continuously recorded multichannel intracranial stereoelectroencephalographic (SEEG) signals before, during and after SE. Results from this analysis indicate trends of progressive reduction of EI over hours up to the onset of SE, mainly at sites of the epileptogenic focus with reversal of those trends upon successful treatment of SE by AEDs. The proposed analytical framework is promising for elucidation of the pathology of neuronal network dynamics that could lead to SE, evaluation of the efficacy of SE treatment strategies, as well as the development of biomarkers for susceptibility to SE.

## Introduction

Status epilepticus (SE) is a life-threatening neurological and medical emergency seen commonly at tertiary care epilepsy centers (1, 2). SE is characterized by recurrent epileptic seizures without recovery of normal brain function between seizures or continuous seizure activity lasting long enough to produce a fixed and enduring condition (3, 4). Out of the 200,000 cases of SE diagnosed each year in the USA, the short-term (30 days) mortality rate reported in the adults ranges between 15 and 30% (2, 5). Although the disease has been reported as early as 718 BC (6), progress in developing new therapy has been slow as the underlying pathophysiological mechanism is poorly understood. Unlike seizures that can self-terminate within minutes, the neural condition of SE can self-perpetuate and self-sustain over hours to days. Both preclinical and clinical studies have demonstrated that seizures that are provoked by infection or induced by stimulants can transition to SE if the initial insult is removed, even in subjects without a known history of epilepsy (7–11). In convulsive SE, the recorded electroencephalographic (EEG) activity of the brain progresses through five visually distinguishable sequential stages that are characterized well in preclinical studies and have also been reported in patients (12, 13). All these studies suggest that SE may not be a continuum of multiple seizures but rather a distinct entity with its own underlying mechanisms.

Over the past two decades, our group as well as other researchers have demonstrated that quantitative analysis of EEG helps characterize parts of the transition of the epileptic brain into SE, namely the interictal (away from seizures), preictal (before seizures), ictal (during seizures), and postictal (after seizures) states (14–17). This analysis not only provides insight into the spatiotemporal dynamics of the epileptic brain but can impact clinical care by allowing long-term prediction of seizures and SE (18–22), as well as interictal localization of the epileptogenic focus (23, 24). By advanced analysis of scalp and intracranial EEG within the framework of nonlinear dynamics, our group first discovered that the epileptogenic focus (zone) progressively, over minutes to hours, entrains normal brain sites into a pathological state of reduced rate of information processing that could mathematically, and thus objectively, help determine preictal periods [see (20) and references therein]. Following self-termination of seizure, there is a postictal disentrainment of the brain sites that were entrained preictally with the epileptogenic focus. We have called this phenomenon of progressive preictal entrainment and postictal disentrainment resetting of the pathology of brain dynamics (25–27). Such a functional role of seizures constitutes a new way of looking into epilepsy as a dynamical disorder and could lead to the development of innovative treatments for epilepsy. Based on these findings, we postulated that (a) subjects can transition to SE if seizures fail to reset the pathology of preictal brain dynamics and (b) successful treatment of SE may help reset this developed pathology of brain dynamics.

In the SE case we herein study, the above postulates seem to be supported. We analyzed for the first time a unique record of SE: a 24-h intracranial, high-density (94 channels) and high sampling frequency (1 kHz), stereoelectroencephalographic (SEEG) recording from a patient long before, during, and long after a 3-h SE episode that he survived. We provide evidence that this patient transitioned to SE after seizures failed to reset the preceding abnormal spatiotemporal dynamics of the focus with the rest of the brain, and that successful treatment of SE reset this pathology. Novel measures of brain dynamics were employed to quantify the route into and out of SE in this patient. The proposed analytical framework and the developed measures shed light on the physiological mechanisms of SE, are promising for evaluation of susceptibility to SE, as well as the evaluation of efficacy of SE treatments and the development of future treatment strategies for SE.

## Materials and Methods

### SEEG Data

A 27-year-old, right-handed male with known medically refractory focal epilepsy that started when he was 14 years old. His epilepsy risk factor included febrile seizures with spinal meningitis during childhood (6 months old) that resolved when he was 1 year old. He was experiencing three seizure subtypes: (1) focal onset seizures without loss of awareness (previously called simple partial) (28) where he reported déjà vu, increased anxiety lasting 10–30 s with frequency 20 per day; (2) focal onset seizures with impaired awareness (previously called complex partial), lip and manual automatism lasting 2–3 min at a frequency of 1–2 times every week; and (3) focal to bilateral tonic–clonic seizures 1–2 times per year. His MRI brain was non-lesional, but 18-fluoro-2-deoxyglucose (18F-FDG) PET-imaged showed right temporal (mesial and lateral) hypometabolism. After scalp EEG monitoring, he underwent right anterior temporal lobectomy at an outside hospital when he was 21 years old. He was seizure free for first 6 months, but all his seizure subtypes gradually returned and progressively increased in frequency. He was then reevaluated for possible second epilepsy surgery at our level-IV epilepsy center (University of Alabama at Birmingham).

**Figure 1:**
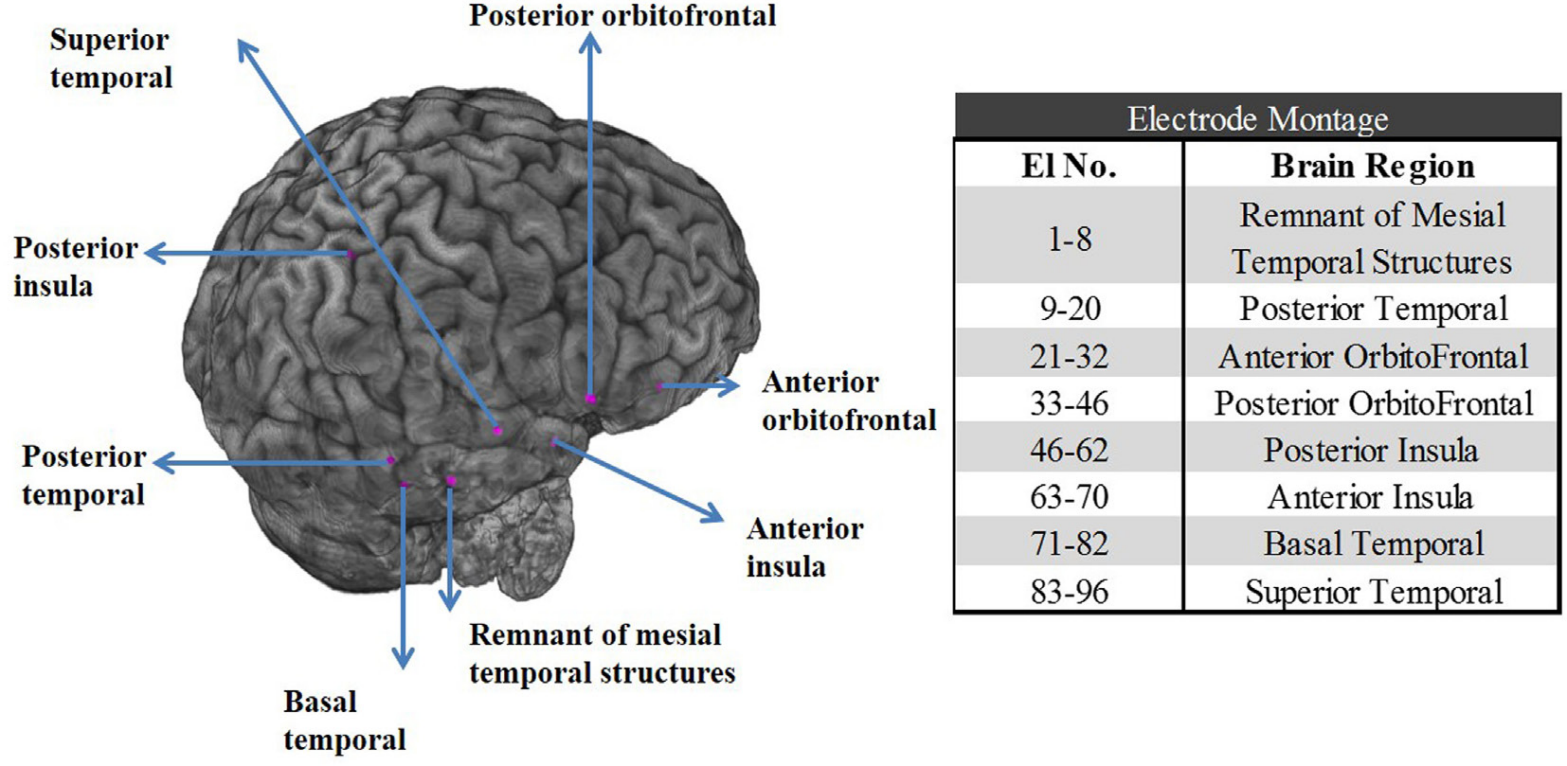
Left panel: Postoperative CT scan merged with preoperative MRI shows the location of the stereoelectroencephalographic electrodes in the right temporal and orbitofrontal structures for patient PT1. Right panel: Table with the electrode contact numbers and the corresponding eight brain regions the electrodes were implanted in.

The patient underwent SEEG monitoring with depth electrodes implanted in the right anterior and posterior insula, right basal temporal, right anterior and posterior orbitofrontal, a remnant of right mesial temporal structures, posterior and superior temporal gyrus. The superior temporal depth electrode was extended medially to include ventrolateral thalamus. Overall, eight depth electrodes with a total of 96 contacts (i.e., 96 channels) were sampled at 1 kHz with a Natus Xltek EEG machine (see the electrode montage in Figure 1).

During his stay in the epilepsy monitoring unit (EMU), his home antiepileptic (AED) medications (clonazepam, calproic acid, oxcarbazepine) were gradually weaned off. Within days after weaning off medications, electroclinical seizures, and subsequently a prolonged (lasting over 3 h) SE was recorded. The SE was of focal onset around the remnant of previously resected mesial temporal structure, progressed gradually to involve insula, lateral temporal structures, and orbitofrontal. The SE spread to the thalamus in the second half of the SE.

Before the onset of SE, patient was awake and alert. The patient initially had no definite clinical correlates which subsequently progressed to bilateral tonic–clonic activities. Throughout the SE, patient was examined periodically by the nurse and EEG technician in the EMU. Examination included assessment of speech (by asking to read a sign board), awareness (by asking about time, place), motor response (by asking to grab a pen), and asking the patient to self-report his feelings during the event. Patient had fluctuating consciousness in the first half of the SE but was lethargic, confused, and had minimal motor response toward the end. Immediately after SE, patient was not oriented to time or place but had appropriate motor response and had no aphasia.

The SE was successfully managed by parenteral valproic acid and two doses of lorazepam. The patient eventually underwent a large resection of temporal lobe structures that included the remnant hippocampus, superior temporal gyrus, and anterior insula [these structures were clinically identified as the seizure onset zone (SOZ)]. Eighteen months postresection the patient is seizure free (Engel’s class I) and remains on AED medication.

Histopathology confirmed chronic neuronal loss, the presence of dysmorphic neurons and extensive gliosis. In particular, immunohistochemical stains for GFAP, neurofilament protein (NFP), and Neu-N neuronal marker were performed on all the tissue blocks. GFAP staining showed mild gliosis in middle temporal gyrus, moderate gliosis in superior temporal gyrus, and marked gliosis in residual hippocampus. Neu-N staining showed mild neuronal losses in hippocampus, middle and superior temporal gyrus. Staining for hyperphosphorylated NFP showed no neuronal somatic staining in middle temporal gyrus, occasional positive cortical cells in superior temporal gyrus, and also a few scattered positive neuronal cells in hippocampus. Appropriate staining was seen in the positive and negative controls. The margin of superior temporal gyrus and the remnant of right hippocampus showed dysmorphic neurons. Anterior insula and margin of middle temporal gyrus showed chronic neuronal loss and astrogliosis.

A 24-h portion of the SEEG (starting 12 h before the onset of SE) was stored and made available for retrospective mathematical analysis. This retrospective case study had institutional IRB approval from the University of Alabama at Birmingham.

### Energy Measure

We employed the Teager Energy (TE) operator (29, 30) to estimate the energy of the SEEG and detect occurring seizures. TE has been used widely for seizure detection, solely or in combination with other measures of EEG characteristics (31–33). TE was applied to band-pass (5–15 Hz) filtered 60 s, non-overlapping consecutive SEEG segments over the duration of the available recording per channel. TE was estimated by:

(1) $$\text{TE}(i)=x^{2}(i)-x\left(i-1\right)\cdot x\left(i+1\right),$$

where *x*(*i*) is the amplitude of the SEEG signal at time point *i*. With a sampling frequency of 1 kHz, 60,000 TE(*i*) values were produced for every 60 s SEEG segment. Then, the median (TE_med_) of those TE values was estimated per 60 s segment generating a TE_med_ temporal profile per electrode site every 60 s. Finally, averaging the TE_med_ profiles across electrodes (spatial averaging) generated the TE_global_ energy profile. We herein employed the TE_global_ measure as a tool for detection of seizures and SE from the SEEG.

### Local Effective Information Inflow Measure

We employed a recently proposed measure of the directional inflow of information a node is experiencing from its communication with the rest of the nodes in the network, the measure of the total effective inflow (TEI) of information. The TEI is estimated from the generalized partial direct coherence (GPDC) that measures the directed (causal) interactions between nodes in a network based on the principle of Granger’s causality (23, 24). We have shown that GPDC outperforms many traditional measures of directed interactions in the frequency domain [e.g., partial coherence, directed coherence, partial directed coherence (PDC), normalized PDC, directed transfer function (DTF), normalized DTF] when the question of localization of the epileptogenic focus is addressed (see (23) and references therein). In particular, from such an analysis of interictal intracranial EEG recordings (23) and interictal magnetoencephalographic (MEG) recordings in respective groups of patients with focal epilepsy (24), we have found that the epileptogenic focus can be accurately localized as the region that most frequently receives the maximum effective inflow (TEI) from other brain regions in the interictal period. We briefly describe our methodology below.

A multivariate autoregressive model $X\left(t\right)=\sum_{\tau =1}^{p}A\left(\tau \right)X\left(t-\tau \right)+\epsilon \left(t\right)$, of order ***p*** = 7 and dimension of vector ***X*** equal to 96, was fit to each of 60 s successive, non-overlapping SEEG segments over the available 24-h SEEG recording from our patient. Components of the vectors ***X*** were the values of SEEG from each of the 96 electrodes at every time point *t*. The coefficients ***A*** (7, 96) of the model were estimated *via* the Vieira-Morf partial correlation method. The GPDC value from site *j* to site *i* was estimated by:

(2) $${\text{GPDC}}_{j\to i}\left(f\right)=\frac{\left|B_{ij}\left(f\right)\right|/\sigma_{ii}}{\sqrt{\sum_{k=1}^{n}{\left|B_{kj}\left(f\right)\right|}^{2}/\sigma_{kk}^{2}}},$$

where $B\left(f\right)=I-\sum_{k=1}^{p}A\left(\tau \right)e^{-i2\pi f\tau }$, and σ*_ii_* is the standard deviation of the generated fitting error from the model at each site *i*. Then, $\sum_{j=1,j\neq i}^{n}{\mathrm{GPDC}}_{j\to i}(f)$ measures the effective information inflow (EI*_i_*(*f*)) to each site *i* from the rest (*n* − 1) of the recording sites at each frequency *f* with resolution of 1 Hz. The EI*_i_*(*f*) values were then averaged over all frequencies in each of three spectral bands: 0.1–50 Hz [low frequency band (LFB)], 70–110 Hz [high frequency band (HFB)], and 130–170 Hz [ultrahigh frequency band (UFB)] producing the TEI*_i_* values per electrode site *i* within specific spectral bands. TEI*_i_* has been proven extremely valuable in localization of the epileptogenic focus from interictal periods (23).

### Global EI and Local EIs

To characterize the brain’s global dynamics through SE, the TEI*_i_* measures of connectivity were averaged across all 96 electrode sites for each 60 s SEEG segment, and the TEI_mean_ (mean) and TEIσ temporal profiles were then calculated every 60 s.

To illustrate the relation over time of the effective local inflow per site versus the effective global inflow, we divided the values of each site’s TEI*_i_* by the values of TEI_mean_ within each 60 s SEEG segment, thus creating a normalized TEI*_i_*_,norm_ profile over time per site *i*. In order to also gain a better insight into the relative position of each site’s TEI*_i_* values compared to the ones at other sites, we ranked the TEI*_i_* values of the *n* = 96 individual sites within each 60 s SEEG segment and plotted the rank of each site *i* over time (TEI*_i_*_,rank_ profiles).

## Results

**Figure 2:**
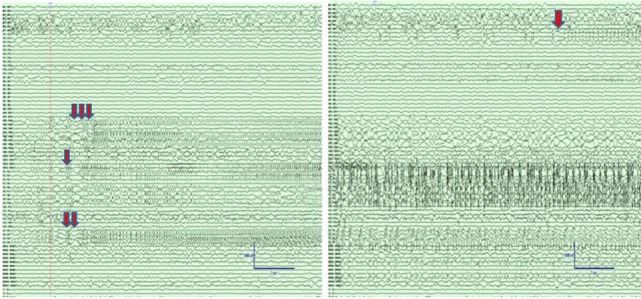
Left panel: Intracranial electroencephalographic (EEG) recording from patient PT1 at the onset of status epilepticus (SE) in the mid insular electrodes (one red arrow) and anterior insular electrodes (two red arrows) followed by propagation to superior temporal gyrus (STG) and orbitofrontal regions (three red arrows). Right panel: Intracranial EEG recording 5 min after the onset of SE with continuous seizure activity in insula and STG, and the onset of spread to the middle temporal gyrus (orange arrow). The EEG records are illustrated with a 10 mm/s time base, sensitivity 50 µV/mm, and filtered between 1 and 100 Hz.

We analyzed a 24-h 96 channel SEEG recording from a patient who had SE while at the EMU and survived following successful treatment with antiepileptic medications. The recording included 8-h pre-SE, 3-h SE, and 13-h post-SE SEEG. Portions of the electroencephalographic manifestation of SE in this patient are shown in Figure 2.

### Detection of SE by Energy measure

**Figure 3:**
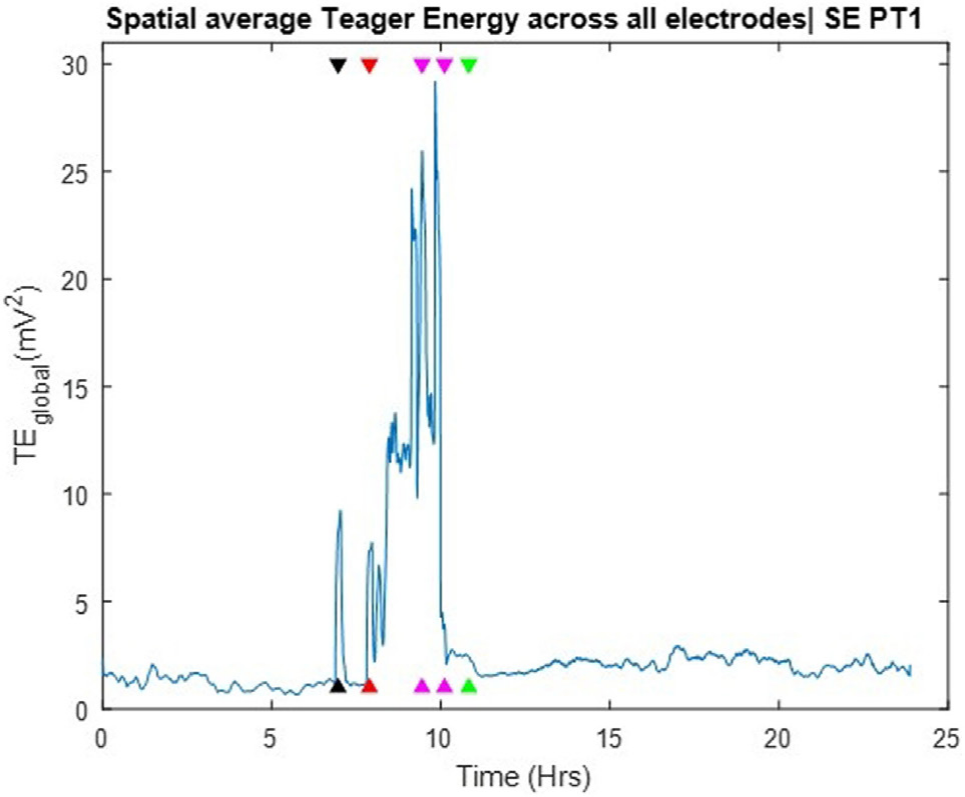
Spatial average of Teager energy across brain sites (TE_global_) over the entire 24 h iEEG recording from PT1. The black arrow, about 7 h into the recording, indicate the timing of a pre-status epilepticus (pre-SE) clinical seizure; the red arrow indicate the onset of SE; the magenda arrows indicate the times of the administered anti-SE medical intervention; the green arrow indicate the clinically assessed onset of recovery from SE. TE_global_ attains high values at the occurrence of the pre-SE clinical seizure as well as at the onset and during most of SE.

The TE values per electrode were averaged across all electrode sites over time and the global Teager energy (TE_global_) profile was estimated from the available 24 h SEEG record and is depicted in Figure 3. About 1 h before the development of SE, the patient had one clinical seizure that was well captured by the occurrence of a prominent (well above the background) peak in the global TE (black arrow in Figure 3). At the onset of SE, and during its 3 h duration, TE_global_ values became prominent again and stayed elevated until the end of the medical interventions (magenta arrows in the figure), after which TE_global_ abruptly assumed low, but still a bit greater than its pre-SE, values over hours (recovery period). Two points are worth noting here: (a) the resetting of TE_global_ values occurred at the end of the medical interventions, that is, about 1 h prior to the clinically assessed end of SE (green arrow). This raises the possibility for use of TE_global_ as an early biomarker of the effectiveness of the administered treatment of SE; (b) the post-SE values of TE_global_ remained elevated compared to its pre-SE values over hours, at least till the end of this recording. This raises the possibility of using the difference between pre and post-SE values of TE_global_ to objectively measure the duration of the recovery period for patients with SE. Finally, it is noteworthy that no pre-SE trends were captured by the TE profiles, that is, issue of warnings for an upcoming SE based on TE_global_ would not have been possible.

### Dynamics of the SE Transition by Information Flow Profiles

**Figure 4:**
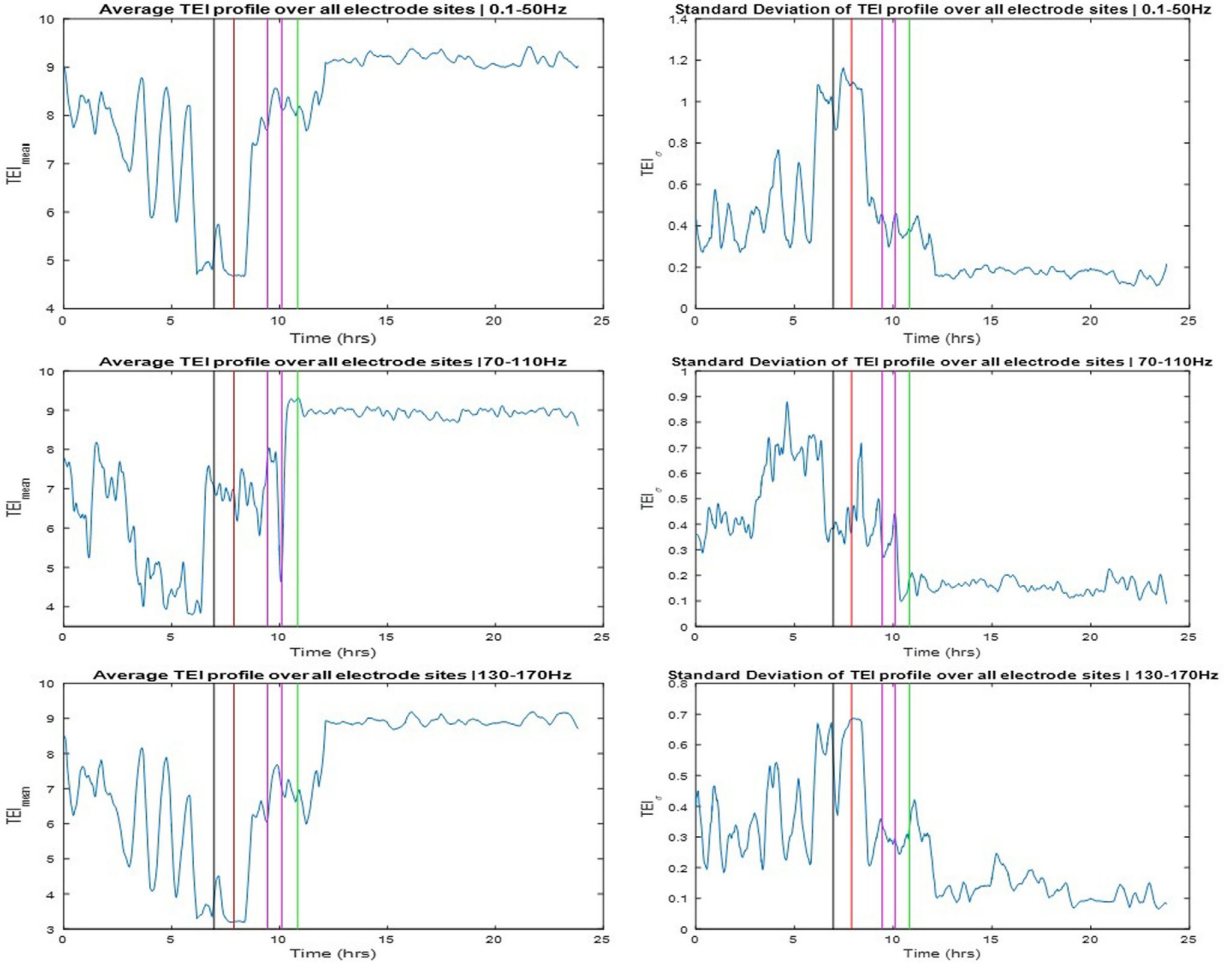
Left panels: Averaged total effective inflow (TEI) values across brain sites over time (TEI_mean_ profiles) for three frequency bands (low frequency band top, high frequency band middle, and ultrahigh frequency band bottom panels—see text for more details). Right panels: Standard deviation of TEI values across brain sites over time (TEIσ profiles) for the same frequency bands. Color coding of the vertical lines same as the one for the arrows in Figure 3.

The TEI profiles and their spatial average (TEI_mean_) and standard deviation (TEIσ) were also estimated over the available 24 h SEEG record and over three different frequency bands: LFB = 0.1–50 Hz, HFB = 70–110 Hz, and UFB = 130–170 Hz. The TEI_mean_ profiles are shown in the left and the TEIσ profiles in the right panels of Figure 4. The TEI_mean_ profiles from LFB and UFB showed a progressively decreasing trend in values up to the onset of SE (red vertical line) with a reverse (increasing) trend thereafter and a further abrupt increase to a higher and steady level post-SE about 1 h after the onset of clinically assessed patient’s recovery (green vertical line). The TEI_mean_ profile from the HFB showed a similarly progressive decrease in values pre-SE almost up to the onset of the pre-SE clinical seizure, with resetting of this trend thereafter to high values during SE and an abrupt further increase in values to a steady higher level right after the administration of the anti-SE AED regiment (magenda lines). It is noteworthy that it was not until approximately 1 h after SE’s end that TEI_mean_ values attained their maximum value in all frequency bands. It is also noteworthy that the variance in TEI values (TEIσ) in all frequency bands (illustrated in the right panels of Figure 4) is higher pre-SE than post-SE, implying the existence of a much more unstable pre-SE state compared to the post-SE state. Finally, it is also noteworthy, from all frequency bands, that a couple of hours after onset of patient’s recovery and assuming resetting of the pathology of dynamics: (a) the TEI_mean_ obtains its highest values, that is, even higher values than the ones 8 h before the onset of SE, implying that the observed downward trend of TEI_mean_ values and the route to SE may have started even earlier than the beginning of the recording and (b) the TEIσ obtains its lowest values, that is, even lower values than the ones 8 h before the onset of SE, again implying that instabilities and the route to SE may have started even earlier than the beginning of this recording.

**Figure 5:**
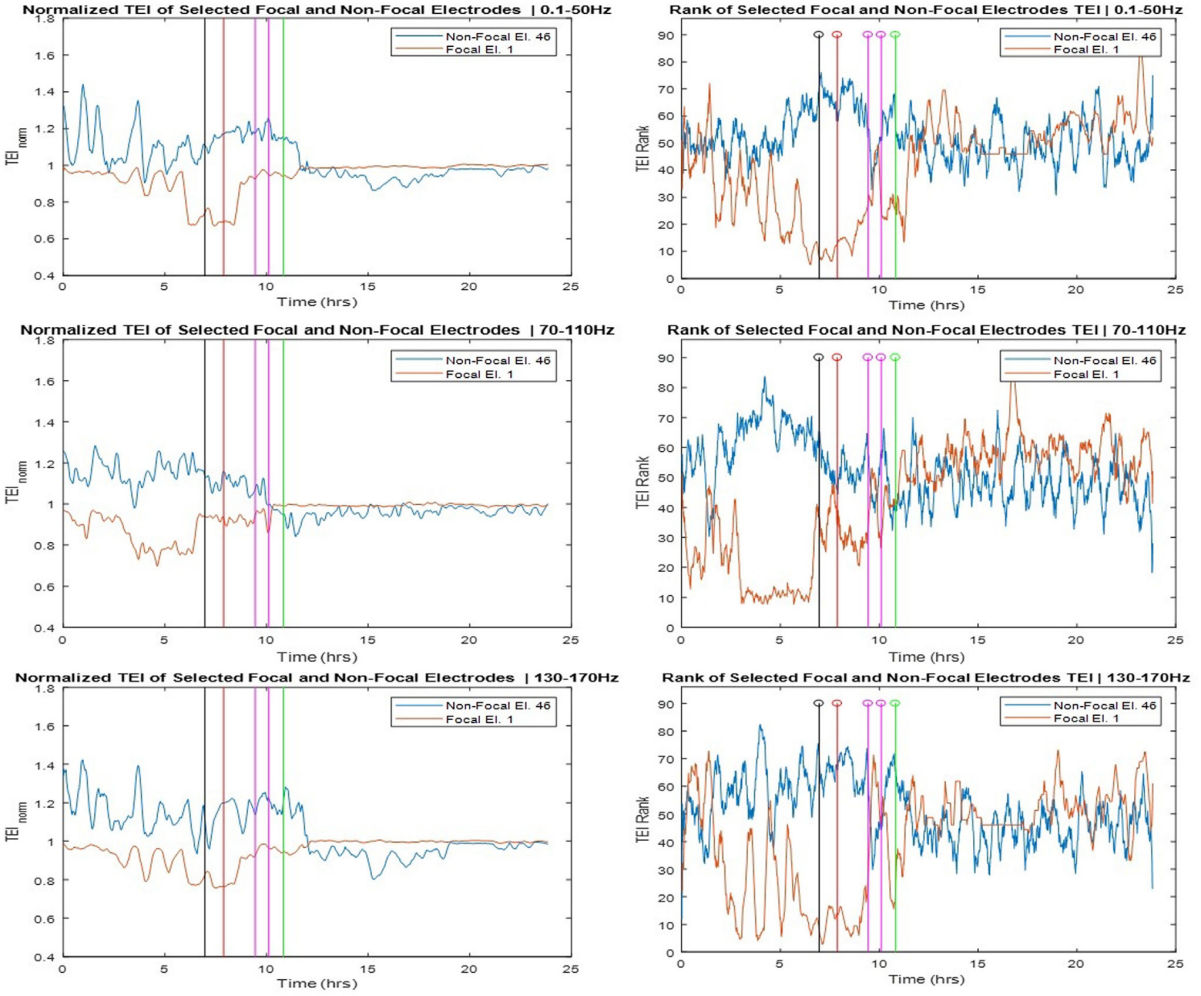
Left panels: Normalized total effective inflow (TEI_norm_) profiles over time at one focal (Elec. 1) and one non-focal (Elec. 46) brain site for three frequency bands (low frequency band top, high frequency band middle, and ultrahigh frequency band bottom panels—see text for more details). Right panels: Rank of the focal and non-focal electrode in terms of their TEI_norm_ values over time for the same three frequency bands.

The SOZ of this patient was determined in a multidisciplinary patient management conference taking in consideration input from several recording modalities including EEG. We labeled electrodes within the SOZ as focal while all the rest as non-focal and examined the relation of the TEI over time between focal and non-focal sites as well as between each individual site and the spatial average (global) flow. Toward this goal, we estimated the ranking of TE values over time (TEI*_i_*_,rank_) at one site *i* versus the rest of the sites, and the normalized TE values over time at one site *i* with respect to the global TE value (TEI*_i_*_,norm_). In Figure 5, we show the TEI*_i_*_,norm_ profiles (left panels) and the TEI*_i_*_,rank_ (right panels) at one focal and one non-focal site. The focal site was within the mesial temporal region, the non-focal site in the posterior orbital frontal and the TE values were estimated in the three frequency bands (LFB, HFB, and UFB). From the left panels of Figure 5, and concentrating on the trends over time of TEI at the focal site with respect to the global EI (focal TEI_norm_ profile), we see that the focal TEI_norm_ values were less than 1 (and hence less than the global TEI values) over the whole pre-SE period (7 h) with this difference increasing dramatically as the onset of SE approaches. After onset of SE, this difference starts to decrease and attains values close to 1 (i.e., focal site inflow approximating the global inflow values) in the post-SE period. The administration of AEDs may have assisted with this increase of the focal inflow. Also from Figure 5, we observe that the trend over time of TEI at the non-focal site with respect to the effective global inflow is opposite from the one at the focal site. If we compare the TEI profiles of the focal and non-focal sites, it is evident that: (a) the EI at the focal site is increasingly becoming smaller than the one at the non-focal site as SE approaches (more evident in LFB) and (b) this difference is reversed post-SE.

The corresponding rank (TEI_rank_) profiles of TEI at the focal and non-focal sites are illustrated in the right panels of Figure 5 and reinforce our observations from the left panels. The rank of the TEI at the focal site drops from about 50 (out of a total of 96) 7 h pre-SE to 10 at the onset of SE and back to 50 post-SE. The rank of the TEI at the non-focal site shows the opposite behavior over time especially in HFB. It is noteworthy that the pre-SE difference between the ranks of TEI at the focal and non-focal sites is reversed post-SE. This resetting is most prominent in the UFB, in which TEI at the focal site is larger than the TEI at the non-focal site (rank of 60 versus 50, respectively) at the beginning of the recording, this trend is reversed within 1.5 h into the recording en route to SE, and reversed again post-SE.

## Discussion

In this case study, we applied novel measures of information flow from analysis of multivariate complex networks in the frequency domain for the study of the transition of the epileptic brain to and out of SE. We employed these measures to analyze the recorded intracranial electrical activity (SEEG) over a 24-h period from a patient who experienced SE and recovered. Using this analytical framework, we provided evidence that the route to SE involved the change of the spatial distribution of the EI of information globally, as well as locally between focal and non-focal sites. In particular, the global TEI measure showed a progressive reduction in its average and increase of its variance across brain sites over hours before the onset of SE, and resetting to its interictal high average and low variance values after the end of SE following the successful anti-SE treatment (Figure 4). Our subsequent analysis at the local level, in terms of TEI profiles at focal and non-focal sites, showed a progressive pre-SE reduction and post-SE resetting mainly at the focal sites, with a pre-SE gap in TEI values between focal and non-focal sites being more evident in the 70–110 Hz frequency band.

The above results, taken cumulatively, suggest that it may be possible to develop TEI-based biomarkers of an impending SE episode, as well as provide feedback on the progress of recovery of a patient in SE. Optimization of the employed frequency band toward this goal would then be necessary, as well as optimization of which and how many focal and non-focal sites to be followed over time. Also, due to the evidence we herein provided that the route to SE may start hours before its clinical onset, longer SEEG recordings before the SE onset may provide additional insight in the physiological mechanisms that lead to SE.

Epilepsy is a chronic neurological disorder believed to be due to existing imbalance between excitation and inhibition in brain’s neural circuit (34, 35). However, static changes within the neural circuit fail to explain the intermittency of seizures as well as the *de novo* SE (i.e., SE occurrence in patients without prior known epilepsy). We have conceptualized in the past that effective feedback to seizure focus from other brain sites may keep the focus under control and allow the epileptic brain to operate more normally interictally (36). Failure of this control mechanism preictally can be detected as a progressive decrease of inflow of information to epileptogenic focal sites allowing them to export their destabilizing signals to the rest of the brain, eventually leading to seizures. The seizures reset this pathology of dynamics by reestablishing the internal feedback control, possibly by ictal release of suitable neurochemical agents. In this study, using GPDC, a measure of directed information flow in coupled networks (23), we showed for the first time that this hypothesis might be true in SE too. In particular, we provided evidence that progressive decrease in EI to the focus characterizes the transition from interictal period to SE and that successful administration of anti-SE AEDs reestablishes the EI to the focus.

Finally, this study was performed on rarely available SEEG data from a patient who underwent SE while at the EMU with implanted EEG electrodes (phase II recording) and needs to be replicated in a larger cohort of patients for further validation. Interinstitutional collaboration to this end would help.

## Conclusion

We provided evidence that transition to SE could be preceded by a gradual, over hours, development of pathology in spatiotemporal dynamics of brain’s electrical activity underlined by the interplay of the epileptogenic focus and controlling normal brain sites, and that successful treatment of SE resets this pathology of brain dynamics. Novel measures of brain dynamics from network analysis in the frequency domain can shed light on this transition and are thus also promising in the evaluation of susceptibility to SE, efficacy of current treatment of SE, and development of future treatment strategies for SE.

## Ethics Statement

Data analysis was performed retrospectively using the EEG that was obtained for clinical reason. The study has approval from the institutional review board (UAB) to perform analysis and publish de-identified data. The patient informed consent is waived in these cases by UAB IRB. There is a collaboration agreement between the two institutes (UAB Epilepsy and Louisiana Tech) for sharing of de-identified patient data and publishing.

## Author Contributions

DP acquired the SEEG data for this study under the supervision of SP. TH performed the analysis of the data under the supervision of LI. All authors contributed equally to the writing of this article.

## Conflict of Interest Statement

The authors declare that the research was conducted in the absence of any commercial or financial relationships that could be construed as a potential conflict of interest.
